# Characteristics and Antioxidant Activity of Fucoidan from *Sargassum hystrix*: Effect of Extraction Method

**DOI:** 10.1155/2022/3689724

**Published:** 2022-04-13

**Authors:** Amir Husni, Nuzulia Izmi, Fatimah Zahra Ayunani, Aprilia Kartini, Naila Husnayain, Alim Isnansetyo

**Affiliations:** ^1^Department of Fisheries, Faculty of Agriculture, Universitas Gadjah Mada, Bulaksumur, Yogyakarta 55281, Indonesia; ^2^Magister in Fisheries Science, Faculty of Agriculture, Universitas Gadjah Mada, Bulaksumur, Yogyakarta 55281, Indonesia

## Abstract

Fucoidan is a bioactive compound of brown seaweed with antioxidant characteristics. This study examined the aftermath of the extraction method on the yield, fucose content, xylose content, sulfate content, total sugar, antioxidant activity, and functional groups of fucoidan from *Sargassum hystrix*. The brown seaweed was extracted using 4 methods, namely, A (0.1 N HCl, room temperature, 24 h), B (2% CaCl_2_, 85°C, 4 h), C (85% ethanol, room temperature, 12 h), and D (0.5% EDTA, 70°C, 3 h). The antioxidant activity testing was carried out using the 2,2-diphenyl-1-picrylhydrazyl (DPPH), Ferric-Reducing Antioxidant Power (FRAP), and Hydroxyl Radical Scavenging Activity (HRSA). The yield for methods of A, B, C, and D was 2.46 ± 0.30, 0.68 ± 0.34, 1.18 ± 0.15, and 0.62 ± 0.25%, with fucose content of 39.97 ± 4.82, 26.72 ± 3.38, 41.08 ± 9.49, and 40.62 ± 8.59%, xylose content of 8.07 ± 0.92, 5.63 ± 0.40, 6.80 ± 0.83, and 7.83 ± 1.83%, and the sulfate content of 11.47 ± 2.20, 15.31 ± 2.47, 30.62 ± 2.76, and 27.80 ± 3.59%. The result indicated the occurrence of a sulfate ester group in the functional group analysis with numerous similarities with the commercial fucoidan. The highest antioxidant activity of fucoidan from *S. hystrix* was found in method C, which was influenced by sulfate levels. Therefore, the extraction method of fucoidan from *S. hystrix* affects the characteristics and antioxidant activity.

## 1. Introduction

The human body comprises trillions of cells that routinely produce free radicals and reactive oxygen species (ROS), which are part of the metabolism process [1]. Oxidative stress occurs because of lack of balance between the production and accumulation of free radicals, which exceeds the number of antioxidants in the body, thereby damaging the cell. According to Werdhasari [2], damages to cells decrease their function and cause several degenerative diseases in humans. Therefore, exogenous antioxidants are needed to overcome and prevent oxidative stress. Exogenous antioxidants can be sourced from synthetic and natural ingredients. Several studies have shown that many natural antioxidants are commonly obtained from plants [3–6]. Additionally, Lailatussifa et al. [7] research mentioned that extracts of brown seaweed are known as the source of natural antioxidants. Antioxidant sources of brown seaweed can come from the presence of bioactive compounds [8] and fucoidan [9].

Fucoidan is a sulfated polysaccharide that originates from various brown seaweed species, and it possesses antioxidant biological activities [10], which help to prevent diseases and ward off free radicals [11]. Fucoidan has been increasingly studied over the years in the pharmaceutical field applications [12], and as functional food additives [13]. The biological activity of fucoidan is associated with its structure, especially the sulfate group attached to the fucoidan monomer [14]. The antioxidant activity of fucoidan brown seaweed *Sargassum* sp. of previous studies includes *Sargassum crassifolium* [15], *Sargassum cristaefolium* [16], *Sargassum hystrix* [17], and *Sargassum muticum* [18]. According to Fernandez et al. [19] and Sinurat and Kusumawati [20], the characteristics and antioxidant activity of fucoidan are affected by algae species, environmental conditions, geographic location, season, and the extraction method used.

Sinurat and Kusumawati [20] researched the extraction of fucoidan from *Sargassum binderi*, using different solvents under varying conditions. However, no study has been carried out to examine the aftermath of the different extraction methods on the characteristics and antioxidant activity of fucoidan from *S. hystrix*. Therefore, this study examined the aftermath of the extraction methods on the characteristics and antioxidant activity of brown seaweed fucoidan from *S. hystrix*.

## 2. Materials and Methods

### 2.1. Materials

*S. hystrix* was obtained from Teluk Awur, Jepara, Central Java, in November 2019. The seaweed was washed and dried for 24 hours in an oven at 60°C [21] before it was cut, grounded, and sieved using a 60-mesh sieve. The initial weight of the powder was examined and stored at a temperature of -20°C before usage.

### 2.2. Fucoidan Extraction

**Figure 1:**
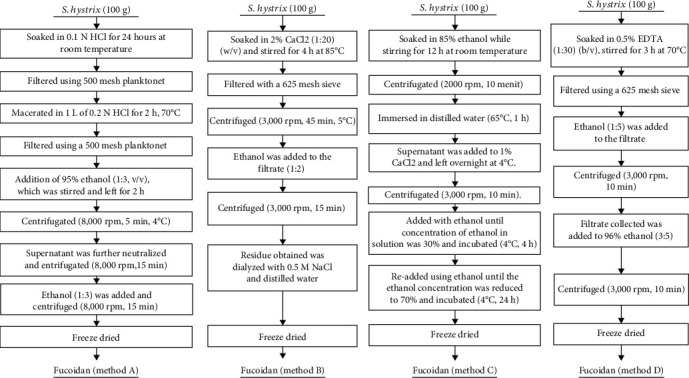
Schematic diagram of four extraction methods of fucoidan from *Sargassum hystrix.*

The schematic diagram of fucoidan extraction is shown in Figure 1.

#### 2.2.1. Method A (0.1 N HCl, Room Temperature, 24 Hours)

This fucoidan extraction method was proposed by Purbomartono et al. [22]. A total of 100 grams of seaweed was soaked in 1 L of 0.1 N HCl, stirred for 24 hours at room temperature, and filtered using a 500-mesh planktonet. The residue was further macerated in 1 L of 0.2 N HCl for 2 hours at 70°C. In addition, the mixture was filtered and evaporated using a 500-mesh planktonet and a rotary evaporator to a volume of 150 mL. This was followed by the addition of 95% ethanol (1 : 3, *v*/*v*), which was stirred and left for 2 hours. The mixture was centrifugated at 8,000 rpm for 5 minutes at 4°C. The supernatant was further neutralized using a 0.5 M NaOH. It was then stirred for 30 minutes at room temperature and centrifugated at 8,000 rpm for 15 minutes. Finally, ethanol (1 : 3) was added to the collected supernatant and centrifuged at 8,000 rpm for 15 minutes, thereby leading to the formation of pellets in the form of a paste, which was dried by freezing.

#### 2.2.2. Method B (2% CaCl_2_, 85°C, 4 Hours)

This method was proposed by Sinurat and Kusumawati [20]. The seaweed powder (100 g) was put into a 2 liter Erlenmeyer and soaked in 2% CaCl_2_ (1 : 20) (*w*/*v*). It was then extracted and stirred with a hot plate stirrer for 4 hours at 85°C. Afterward, it was filtered with a 625-mesh sieve with the filtrate collected and centrifuged at 3,000 rpm for 45 minutes at 5°C. The supernatant was collected in a jar while the sediment was disposed. In addition, ethanol was added to the filtrate at a ratio of 1 : 2, while the precipitate was dissolved in water and centrifuged at 3,000 rpm for 15 minutes. Consequently, the residue obtained was dialyzed with 0.5 M NaCl and distilled water and dried with a freeze dryer.

#### 2.2.3. Method C (85% Ethanol, Room Temperature, 12 Hours)

Palanisamy et al. [23] modified the previously proposed extraction method to obtain method C. A total of 100 grams of seaweed powder were immersed in 85% ethanol and mechanically stirred at room temperature for 12 hours. Furthermore, the mixture was centrifugated for 10 minutes at 2,000 rpm, with the residue collected, and dried at room temperature till it was reconverted into a powdered form. In addition, the dried residue was immersed in distilled water at a temperature of 65°C for 1 hour before it was centrifuged at 3,000 rpm for 10 minutes. The supernatant was added to 1% CaCl_2_ and left overnight at a temperature of 4°C. The mixture was radiated from a central point at 3,000 rpm for 10 minutes. Then, the supernatant was added with 96% ethanol until the concentration of ethanol in 30% solution and incubated at 4°C for 4 hours. The solution was readded using ethanol until the ethanol concentration was reduced to 70% and incubated at 4°C for 24 hours. A sediment's appearance is followed by filtering the solution using a filter paper to collect the residue, which was dried using a freeze dryer.

#### 2.2.4. Method D (0.5% EDTA, 70°C, 3 Hours)

This method was proposed by Zhao et al. [24]. A total of 100 grams of seaweed powder were immersed in 0.5% EDTA (1 : 30, *w*/*v*) and stirred in a water bath for 3 hours at 70°C. The solution was chilled at room temperature and filtered using a 625-mesh sieve. Ethanol (1 : 5) was added to the filtrate and then centrifuged at 3,000 rpm for 10 minutes. The filtrate collected was added to 96% ethanol (3 : 5) and centrifuged at 3,000 rpm for 10 minutes. The pellets were then freeze-dried while the sediments were discarded.

### 2.3. Fucoidan Yield

The fucoidan yield was collected by dividing the weight of fucoidan by the weight of dry seaweed powder and multiplying it by 100.

(1) $$\begin{array}{c} \text{Yield}=\frac{\text{Weight of fucoidan}}{\text{Weight of dry seaweed }}\times 100\%. \end{array}$$

### 2.4. Functional Group Analysis

This analysis was tested with a Fourier Transform Infrared (FT-IR) Spectrophotometer (Perkin-Elmer 577) to examine the form of functional groups in fucoidan from *S. hystrix*. The process was conducted using the method proposed by Sinurat and Kusumawati [20] with 2 mg of the total sample crushed to homogeneous using 200 mg of potassium bromide (KBr). In addition, the powdered mixture was turned into thin and transparent tablets at a pressure of 7,000 Pa. The sample was further placed in a simple pan to determine the infrared spectrum records at a wavelength of 4,000-500 cm^−1^. Subsequently, the commercial fucoidan from *M. pyrifera* (Sigma-Aldrich, Product of Australia), which was used as the standard, was given a similar treatment as the sample.

### 2.5. Total Sugar

The total sugar content was determined using the phenol-sulfuric acid test based on the method proposed by Dubois et al. [25]. The sugars were tested by preparing fucose (100, 200, 300, 400, and 500 ppm) and xylose (5, 10, 15, 20, and 25 ppm) standard solutions, using a 1,000 ppm sample solution of fucoidan from *S. hystrix*. The phenol-sulfate test was conducted by adding 2.5 mL of concentrated H_2_SO_4_ to each solution and shaken till the mixture became homogeneous. It was then soaked in ice for 20 minutes, after which 0.5 mL (5%) of phenol was added and shaken till a homogeneous mixture was obtained, which was further soaked in ice for 30 minutes. A UV-VIS spectrophotometer was utilized to measure the absorbance of all samples, while the fructose and xylose standards were determined at a wavelength of 490 nm and 480 nm, respectively.

### 2.6. Sulfate Content

The Dodgson and Price [26] BaCl_2_-gelatin method was used in this research to analyze the sulfate content of fucoidan from *S. hystrix*. A homogenous solution was obtained by dissolving 100 mL of aquabides and 0.5 g gelatin in a hot plate stirrer at temperatures ranging between 60 and 70°C. Furthermore, 0.5 g of BaCl_2_ was added to the solution and left for 24 hours at a temperature of 4°C. The sample was also processed by dissolving 6 mg of fucoidan from *S. hystrix* in 2 mL of 3.5 N HCl and stirred till homogeneous. Furthermore, it is centrifugated to ensure that only the supernatant is collected. The commercial fucoidan solution of *M. pyrifera* was given a similar treatment as the sample. Standard K_2_SO_4_ at concentrations of 200, 400, 600, 800, and 1,000 ppm were prepared. Moreover, the sample solution, 3% TCA, and BaCl_2_-gelatin were mixed in a cuvette at a successive ratio of 200, 600, and 300 *μ*L. The mixture was manually stirred and left for 15 minutes, after which a UV-VIS spectrophotometer was used to measure the absorbance at a wavelength of 360 nm. Furthermore, the standard K_2_SO_4_ and the commercial fucoidan from *M. pyrifera* solutions were given similar treatment.

### 2.7. Radical Scavenging Activity (RSA) 2,2-Diphenyl-2-Picrylhydrazyl (DPPH)

The RSA DPPH antioxidant activity test was conducted according to the modified method proposed by Zaranappa et al. [27]. A sample of fucoidan *S. hystrix* and commercial fucoidan from *M. pyrifera* (500 to 4,000 ppm) was prepared using distilled water and allowed to homogenize. Furthermore, 0.76 mM DPPH solution was prepared by dissolving 3 mg of DPPH powder in 10 mL distilled water and stored at a temperature of 4°C for 24 hours. The solution was further incubated at room temperature for 30 minutes with a UV-VIS spectrophotometer used to analyze the absorbance at a wavelength of 517 nm. In addition, the antioxidant was represented in accordance with the inhibition percentage of IC_50_, as follows:

(2) $$\begin{array}{c} \text{Inhibition activity }\left(\%\right)=\frac{\left(C-D\right)-\left(A-B\right)}{\left(C-D\right)}\times 100, \end{array}$$

where *A* is the sample (800 *μ*L sample + 200 *μ*L 0.76 mM DPPH), *B* is the control sample (800 *μ*L sample + 200 *μ*L distilled water), *C* is the negative control (800 *μ*L distilled water + 200 *μ*L 0.76 mM DPPH), and *D* is the blank (800 *μ*L distilled water).

### 2.8. Ferric-Reducing Antioxidant Power (FRAP)

The FRAP test was conducted using the Clarke et al. [28] model, which reduces Fe^3+^ to Fe^2+^ using a spectrophotometer at a wavelength of 595 nm. The changes were analyzed to determine the formation of a blue color in the solution. Furthermore, 0.775 g of sodium acetate trihydrate (CH_3_COON·3H_2_O) was added to 4 mL of concentrated acetic acid which was dissolved in water to determine the acetate buffer solution with pH 3.6. The buffers were stored as stock solutions at a temperature of 4°C with 0.15 g of TPTZ in 40 mM HCl dissolved in 50 mL of distilled water to obtain 10 mM of 2,4,6-tripyridyl-s-triazine (TPTZ). Furthermore, 0.828 mL of concentrated HCl was dissolved in 250 mL of distilled water and a TPTZ solution stored at 4°C for 24 hours to obtain 40 mM HCl. A solution of 20 mM FeCl_3_·6H_2_O was also prepared by dissolving 0.54 g of FeCl_3_·6H_2_O in 100 mL of distilled water for 24 hours at a temperature of 4°C before usage. A FRAP reagent was also prepared by mixing 25 mL of acetate buffer, 2.5 mL of TPTZ, FeCl_3_·6H_2_O solutions (10 : 1 : 1), and 100 mL of distilled water. Furthermore, the standard FeSO_4_·7H_2_O (10,000 *μ*M) solutions were produced by dissolving 2.78 g FeSO_4_·7H_2_O in 1,000 mL distilled water, before serially diluting to attain 50, 100, 150, 200, 250, and 300 ppm concentrations. This was followed by preparing sample solutions from *S. hystrix* (1,000 ppm) and commercial fucoidan (1,000 ppm). Furthermore, a 20 *μ*L solution was added to 150 *μ*L of FRAP reagent in a 96-well microplate using a UV-VIS spectrophotometer at a wavelength of 595 nm (Multiple Go). The standard FeSO_4_ solution and commercial fucoidan were also given similar treatment.

### 2.9. Hydroxyl Radical Scavenging Activity (HRSA)

The HRSA test was conducted using the Zhao et al. [24] proposed method. A total of 0.1 mL of fucoidan samples at concentrations of 250, 500, 1000, 2500, and 5000 ppm was added to a solution of 1 mL 9 mM FeSO_4_, 1 mL 0.3% of H_2_O_2_, and 0.5 mL 9 mM salicylic acid. The mixture was dissolved in 5 mL of distilled water. It was then incubated at 37°C for 30 minutes. A UV-VIS spectrophotometer with a wavelength of 510 nm was used to determine the absorbance. The percentage of HRSA and IC_50_ represented the antioxidant activity. The following formula was used to calculate the HRSA percentage.

(3) $$\begin{array}{c} \text{HRSA}\left(\%\right)=\frac{\left(A0-\left(A1-A2\right)\right)}{A0}\times 100, \end{array}$$

where *A*0 is the absorbance of a sample, *A*1 is the absorbance of control, and *A*2 is the absorbance of reagent.

### 2.10. Total Antioxidants

The total antioxidant activity test was conducted using the method proposed by Salma et al. [29]. A total of 0.023 g of fucoidan *S. hystrix* was mixed with 25 mL water and then produced 3 mL of the reagent (a mixture of 0.6 M sulfuric acid, 28 mM sodium phosphate, and 4 mM ammonium molybdate at a ratio of 150 : 7 : 1). The fucoidan solution and the reagent were mixed till it was homogenized and incubated for 90 minutes at 5°C, after which the mixture was cooled and then vortexed. A spectrophotometer with a wavelength of 695 nm was used to measure the absorbance. Commercial fucoidan from *M. pyrifera* was given a similar treatment as the sample. Vitamin C was used as a standard curve. Therefore, the total value of antioxidant capacity obtained was denoted in mg of the Ascorbic Acid Equivalent (AAE)/g. The following formula was used to calculate the total antioxidant capacity:

(4) $$\begin{array}{c} \text{Total antioxidant }\left(\text{mg AAE}/\text{g sample}\right)=\frac{\left(a\times V\right)/1000}{G}, \end{array}$$

where *a* is the concentration of vitamin C in the test sample (mg/L), *V* is the total volume of test solution (mL), *G* is the weight of extract (g), and 1000 is the conversion factor to total volume of solution (mL).

### 2.11. Data Analysis

The standard deviation (*n* = 3) of all the values was expressed. The data was processed using Excel 2013 and Statistical Package for Social Sciences (SPSS) version 20.0 for Windows (Microsoft Windows, Inc). Normality was determined using the Kolmogorov-Smirnov test. However, assuming the data was normally distributed, there is a need to carry out the parametric analysis using the LSD and Duncan tests. When the data are not normally distributed, a nonparametric analysis is determined using the Kruskal-Wallis and Mann-Whitney tests.

## 3. Results and Discussions

### 3.1. Fucoidan Yield

Fucoidan yield is the ratio of the weight of its extract to the weight of dry seaweed flour expressed in percent (%) [20]. The yields of fucoidan extracted from *S. hystrix* brown seaweed using methods A, B, C, and D are 2.46 ± 0.30, 0.68 ± 0.34, 0.62 ± 0.25, and 1.18 ± 0.15%, respectively, as shown in Table 1. The yield of fucoidan from *S. hystrix* extracted using method C was lower than in methods A, B, and D. This is because ethanol (method C) which has two kinds of polarities, namely, the OH (polar) and the alkyl (nonpolar) groups, was used. Therefore, there is a possibility that some fucoidan dissolved in the polar group, thereby leading to a low yield. There was an insignificant difference between C and B because the solvents (CaCl_2_ and ethanol) used tend to remove components that are insoluble in acidic solvents. In addition, CaCl_2_ specifically deposits alginate, thereby affecting the purity of fucoidan extract, which results in low yields [20].

Fucoidan from *S. hystrix* extracted using methods A and D produced higher yields than others. This is due to the acid solvents (HCl and EDTA), which led to the degradation of the seaweed polymer chains [30]. HCl solvent produces more yield than EDTA because it is a strong acid. According to Puspantari et al. [31], fucoidan extraction using weak acids produces a lesser yield.

### 3.2. Total Sugar

The extraction methods used on the fucose content were derived from *S. hystrix* as shown in Table 1. Fucoidan from *S. hystrix* extracted using method C yielded higher fucose. This is due to the use of ethanol, which specifically precipitates the fucoidan with the help of distilled water during the extraction process. In addition, fucose is soluble in distilled water. According to Sinurat and Maulida [15], fucose is the main component of fucoidan; therefore, a higher content indicates that the extract was pure. The highest fucose content was in method C. Although it has a lower yield, fucoidan *S. hystrix* extracted with ethanol solvent has a greater level of purity than other solvents. Because ethanol acted especially to precipitate fucoidan, the ethanol solvent yields a high fucose content, and the extraction process was aided by the use of distilled water because fucose was very soluble in distilled water. Fucose was the major component of fucoidan, according to Sinurat and Maulida [15]; hence, the higher the fucose level, the purer the fucoidan extract. The use of methods A and D yielded lesser content than C. This is because the acid solvent used during the extraction process attracts fucoidan and also eliminates alginate. However, the extract contains alginic acid [20]. Meanwhile, method B produced a lesser fucose content than the other procedures because CaCl_2_ solvent was used. According to Sinurat and Kusumawati [20], CaCl_2_ precipitates alginate, thereby yielding low fucoidan.

According to Lim et al. [32], the main monomer of fucoidan is fucose and contains other monosaccharides such as xylose. The extraction of the xylose content from *S. hystrix* is shown in Table 1. The xylose content extracted from *S. hystrix* fucoidan using methods A, B, C, and D is 8.07 ± 0.92, 5.63 ± 0.40, 6.80 ± 0.83, and 7.83 ± 1.83%, respectively. On the contrary, the xylose contained in the commercial fucoidan from *M. pyrifera* was 8.07 ± 6.79%. Subsequently, the xylose content of the four fucoidan *S. hystrix* and commercial fucoidan from *M. pyrifera* is insignificantly different (*p* > 0.05).

The extraction methods used on the total sugar content were obtained from the fucoidan from *S. hystrix* as shown in Table 1. Fucoidan from *S. hystrix* extracted using method B has the least amount of sugar, 26.72 ± 3.74%, compared to A, C, and D. The total sugar content obtained using method B was significantly different (*p* < 0.05) from the commercial fucoidan from *M. pyrifera*. Conversely, fucoidan extracted using methods A, C, and D were insignificantly different (*p* > 0.05) compared to the total sugar obtained from the commercial fucoidan from *M. pyrifera*.

### 3.3. Sulfate Content

The effect of the extraction method on the sulfate content derived from *S. hystrix* is shown in Table 1. Method C (30.62 ± 2.75%) produced higher sulfate content than methods A (11.47 ± 2.20%) and B (15.31 ± 2.47%). Higher sulfate groups containing fucoidan manifested stronger antioxidants [33]. The high sulfate content was due to the use of ethanol (method C), which indicates the purity of the fucoidan compound. Ethanol is a good solvent for extracting secondary metabolites because it has the ability to penetrate the cell walls of seaweed without degrading the sulfates [34]. Furthermore, because fucoidan *S. hystrix* has a high sugar content, the sulfate content was similarly proportional to the sugar content; the higher the sugar, the more sulfate ions were bound to the sugar. The low sulfate concentration of fucoidan extracted with HCl solvent may be owing to the strong acid solvent destroying sulfate as well as extracting fucoidan. While the sulfate content of fucoidan extracted with EDTA solvent was better than that extracted with HCl solvent, it was still lower than that extracted with ethanol solvent. This could be due to the fact that EDTA solvent was a weak acid, allowing it to extract fucoidan while maintaining its structure [20]. Fucoidan extracted using CaCl_2_ solvent has a relatively low sulfate level compared to ethanol and EDTA. This could be due to the ability of CaCl_2_ to bind sulfate. Ca^2+^ ions in CaCl_2_ can attach to sulfate ions (SO_4_^2-^) to create CaSO_4_, which causes the sulfate content to be transported away by CaCl_2_ [35].

### 3.4. Functional Group Analysis

**Figure 2:**
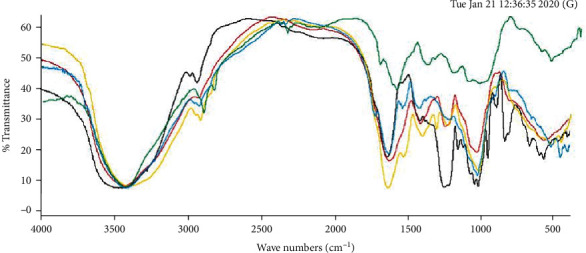
FT-IR spectra of fucoidan from *S. hystrix* (method A: blue line; method B: green line; method C: red line; method D: yellow line) and commercial fucoidan *M. pyrifera* (black line).

The functional groups of fucoidan compound extracts were determined using FT-IR analysis. This involves carrying out infrared absorbance testing on a compound to distinguish its functional groups based on the absorbance pattern [36]. The FT-IR spectra of the four fucoidans from *S. hystrix* and commercial fucoidan are shown in Figure 2. Although there are several functional groups in commercial fucoidan, they have certain similarities, which are not present in *S. hystrix* fucoidan.

Fucoidan from *S. hystrix* (3434.22 cm^−1^, 1631.98 cm^−1^, 1038.46 cm^−1^, 579.25 cm^−1^, and 1252.70 cm^−1^) and commercial fucoidans (3453.96 cm^−1^, 1645.71 cm^−1^, 1029.64 cm^−1^, 580.73 cm^−1^, and 1260.64 cm^−1^) have similar wavelengths. These indicate that several functional groups are present in the compounds. This also includes the absorption area, which has a wavelength of 3453.96 cm^−1^ in commercial fucoidan and 3434.22 cm^−1^ in fucoidan from *S. hystrix* thereby showing the hydroxyl (OH) functional group [10]. Conversely, an absorption area with a wavelength of 1645.71 cm^−1^ and 1631.98 cm^−1^ in the commercial fucoidan and fucoidan from *S. hystrix*, respectively, shows the presence of a carbonyl group (C = O). On the contrary, an absorption area of approximately 1600 cm^−1^ indicates the presence of uronic acid in both compounds [37].

The absorption area with a wavelength of relatively 1000 to 1300 cm^−1^ indicates the presence of sulfate groups in the compounds, for example, wavelengths of 1029.64 cm^−1^ and 1260.64 cm^−1^ in commercial fucoidan and 1038.46 cm^−1^ and 1252.70 cm^−1^ in fucoidan from *S. hystrix*. According to Sinurat and Kusumawati [20], the sulfate ester, a functional group in the absorption area with a wavelength of approximately 1200 cm^−1^, is a characteristic of fucoidan compounds. This shows that the *S. hystrix* extract sample is a fucoidan. However, several functional groups in commercial fucoidan are absent in the *S. hystrix* fucoidan, namely, the absorption area with a wavelength of 1166.97 cm^−1^ indicates sulfate ester, while a wavelength of 851.44 cm^−1^ implies sulfated polysaccharides (COS), which strengthens the characteristics of these compounds [20]. In addition, the absorption area with a wavelength of 2943.27 cm^−1^ signifies the presence of a C-6 group from the paranoid ring [23]. In fucoidan from *S. hystrix*, an absorption area with a wavelength of 1420.39 cm^−1^ indicates the presence of a methyl (CH_3_) functional group [10].

### 3.5. Antioxidant Activity

#### 3.5.1. Radical Scavenging Activity DPPH

The DPPH activity of fucoidan extracted using methods A, B, C, and D had an IC_50_ of 4.336, 5.409, 5.616, and 2.200 ppm, respectively. Conversely, the commercial fucoidan from *M. pyrifera* had an IC_50_ of 1.634 ppm (Table 2). The DPPH activity of fucoidan from *S. hystrix* extracted using method C showed the least activity. This is because it was extracted with ethanol (method C), which has a lesser primary antioxidant activity than the secondary. The RSA DPPH method is used for determining the primary antioxidant activity. Therefore *S. hystrix* shows low antioxidant activity when it was tested with this method [38]. Fucoidan from *S. hystrix* extracted with ethanol solvent is thought to have formed hydrogen bonds between the hydroxyl groups, which act as electron donors in the primary antioxidant mechanism [39]. Unfortunately, when the hydroxyl groups are unable to donate electrons to free radicals, it results in low antioxidant activity.

#### 3.5.2. Ferric-Reducing Antioxidant Power (FRAP)

The effect of the extraction method of fucoidan from *S. hystrix* on the FRAP value is shown in Table 2. Based on this, the antioxidant activity realized from method C provided higher values than A and B. This was due to the fact that the fucoidan was extracted with HCl (method A) and CaCl_2_ (method B) solvents, which have a lesser sulfate content. According to Sinurat and Maulida [15], the antioxidant activity is directly proportional to the fucoidan's sulfate content. Therefore, an increase in the sulfate content causes an increase in antioxidant activity.

#### 3.5.3. Hydroxyl Radical Scavenging Activity (HRSA)

The effect of the extraction method of fucoidan *S. hystrix* on HRSA values is shown in Table 2. Fucoidan from *S. hystrix* extracted using methods A, B, C, and D had IC_50_ HRSA 2312.51 ± 362.15, 1693.97 ± 515.07, 849.37 ± 67.54, and 2360.07 ± 536.93 ppm, respectively, while the commercial fucoidan IC_50_ was 1906.39 ± 537.58 ppm. Based on these results, fucoidan extracted using method C had the least IC_50_ value (highest antioxidant activity). Conversely, method D had the highest IC_50_ value (least antioxidant activity). This was because method C has higher sulfate content than D. According to Wang et al. [40], sulfate content in fucoidan is related to the level of bioactivity. Therefore, the higher the sulfate content in the fucoidan extracts, the higher the antioxidant activity [31]. According to the IC_50_ value, *S. hystrix* fucoidan with ethanol solution has an IC_50_ value of 849.37 ± 67.54 ppm, which is lower than the commercial IC_50_ of fucoidan value of 1906.39 ± 537.58 ppm. This suggested that fucoidan *S. hystrix* has a stronger antioxidant activity than commercial fucoidan, despite the fact that statistical testing shows that the IC_50_ values of the two samples were not substantially different since *p* > 0.05. Although *S. hystrix* fucoidan has a lower sulfate level, it has stronger antioxidant activity than commercial fucoidan. It is likely that other components in the fucoidan extract play a role in its bioactivity besides sulfate. This is because the bioactivity of fucoidan compounds is influenced by three factors: chemical structure, phenol content, and sulfate content [10]. Other secondary metabolites found in brown seaweed include phenolic compounds, flavonoids, alkaloids, glycosides, tannins, and steroids, all of which are assumed to have antioxidant properties [39]. So it is probable that other secondary metabolites besides fucoidan exist in the fucoidan extract of *S. hystrix* and influence the strong antioxidant activity. Fucoidan molecules, on the other hand, have a relatively high bioactivity when compared to other secondary metabolites. This is due to the heterocyclic structure of fucoidan, which is made up of carbon atoms bound to sulfate ions. The sulfate ion can boost electron of fucoidan density, allowing it to act as an electron donor [40].

### 3.6. Total Antioxidants

The effect of the extraction method of fucoidan from *S. hystrix* on the total antioxidant values is shown in Table 2. The total antioxidants from the fucoidan extracted using methods A and B were significantly lower than the commercial fucoidan (*p* < 0.05) due to its higher sulfate content (Table 1). According to Nurhidayati et al. [41], the higher the sulfate content in fucoidan, the higher its ability to reduce free radicals. The total antioxidant value of fucoidan extracted using C and D methods was higher than commercial fucoidan, although insignificantly different (*p* > 0.05). This is not in line with the higher sulfate content of commercial fucoidans, which increases bioactivity. There is a possibility that other compounds besides the sulfate content in the fucoidan were extracted using methods C and D, which act as antioxidants. According to Alboofetileh et al. [10], three factors, namely, chemical structure, phenol, and sulfate contents, affect fucoidan compounds' bioactivity. Apart from the fucoidan compounds, brown seaweed also has other secondary metabolite compounds such as phenolics, flavonoids, alkaloids, glycosides, tannins, and steroids, which are believed to possess antioxidant activity [39]. Therefore, the fucoidan extracted using either method C or D has certain compounds that act as antioxidants.

## 4. Conclusion

The extraction methods of fucoidan from *Sargassum hystrix* affect its characteristics and antioxidant activity. Fucoidan from *S. hystrix* extracted using method A (0.1 N HCl, room temperature, 24 hours) had the highest yield. Subsequently, the highest sulfate content and antioxidant activities were discovered in method C (85% ethanol, room temperature, 12 hours). The highest antioxidant activity of fucoidan from *S. hystrix* is from method C which secondary antioxidants were found to be greater in fucoidan *S. hystrix* extracted with ethanol solvent than primary antioxidants. It has been demonstrated that the DPPH method, which measures primary antioxidant activity, has low activity, whereas the FRAP method, which measures secondary antioxidant activity, has a fairly high antioxidant activity, despite the fact that the FRAP method's antioxidant activity is lower than commercial fucoidans. Similarly, the HRSA method and the total antioxidant approach, which both measure total antioxidant activity, including primary and secondary, both indicate high activity. The antioxidant activity of fucoidan from *S. hystrix* was influenced by its sulfate content.

## Figures and Tables

**Table 1:** The extraction methods on fucoidan yield, total sugar, xylose, fucose, and sulfate contents derived from *S. hystrix.*

Extraction methods	Yield	Total sugar (%)	Xylose (%)	Fucose (%)	Sulfate (%)
A	2.46 ± 0.30^a^	48.68 ± 4.82^a^	8.07 ± 0.92^a^	39.93 ± 4.82^a^	11.47 ± 2.20^c^
B	0.68 ± 0.34^bc^	26.72 ± 3.74^b^	5.63 ± 0.40^a^	21.08 ± 3.38^b^	15.31 ± 2.47^c^
C	0.62 ± 0.25^c^	47.88 ± 10.03^a^	6.80 ± 0.83^a^	41.08 ± 9.49^a^	30.62 ± 2.75^b^
D	1.18 ± 0.15^b^	48.45 ± 10.38^a^	7.83 ± 1.83^a^	40.62 ± 8.59^a^	27.80 ± 3.59^b^
Cf	—	61.74 ± 11.72^a^	8.07 ± 6.79^a^	53.68 ± 10.97^a^	44.36 ± 4.47^a^

Note: A (0.1 N HCl, room temperature, 24 h), B (2% CaCl_2_, 85°C, 4 h), C (85% ethanol, room temperature, 12 h), D (0.5% EDTA, 70°C, 3 h), and Cf (commercial fucoidan). ^a-c^The same letter in the same column shows an insignificant difference (*p* > 0.05).

**Table 2:** Effect of the extraction method on the antioxidant activity derived from the fucoidan from *Sargassum hystrix* (DPPH, FRAP, HRSA, and total antioxidants).

Extraction methods	Antioxidant activity			
	DPPH (IC_50_, ppm)	FRAP (*μ*M/g)	HRSA (IC_50_, ppm)	Total antioxidant (*μ*g AAE/mg)
A	4335.57 ± 1676.74^b^	172.76 ± 6.47^a^	2312.51 ± 362.15^a^	112.41 ± 18.59^b^
B	5408.75 ± 1531.53^bc^	90.40 ± 5.28^bc^	1693.97 ± 515.07^b^	140.81 ± 28.11^b^
C	5616.75 ± 1812.02^bc^	87.90 ± 9.76^b^	849.37 ± 67.54^c^	409.99 ± 83.20^a^
D	2200.23 ± 1014.77^c^	71.38 ± 6.14^bc^	2360.07 ± 536.93^a^	374.82 ± 9.74^a^
Cf	1634.55 ± 409.72^c^	60.40 ± .14^c^	1906.39 ± 537.58^b^	341.01 ± 9.37^a^

Note: A (0.1 N HCl, room temperature, 24 h), B (2% CaCl_2_, 85°C, 4 h), C (85% ethanol, room temperature, 12 h), D (0.5% EDTA, 70°C, 3 h), and Cf (commercial fucoidan). ^a-c^The same letter in the same column shows an insignificant difference (*p* > 0.05).

## Data Availability

The data used to support the findings of this study have been deposited in the Universitas Gadjah Mada repository (http://etd.repository.ugm.ac.id/penelitian/detail/186068;http://etd.repository.ugm.ac.id/penelitian/detail/186611;http://etd.repository.ugm.ac.id/penelitian/detail/190909;http://etd.repository.ugm.ac.id/penelitian/detail/186610).

## References

[1] Sinaga F. A. (2016). Stress oksidatif dan status antioksidan pada aktivitas fisik maksimal. *Jurnal Generasi Kampus*.

[2] Werdhasari A. (2014). Peran antioksidan bagi kesehatan. *Jurnal Biotek Medisiana Indonesia*.

[3] Chaves N., Santiago A., Alias J. C. (2020). Quantification of the antioxidant activity of plant extracts: analysis of sensitivity and hierarchization based on the method used. *Antioxidants*.

[4] Ghosh S., Rana D., Sarkar P. (2022). Ecological safety with multifunctional applications of biogenic mono and bimetallic (Au-Ag) alloy nanoparticles. *Chemosphere*.

[5] Roy S., Rhim J. W. (2020). Curcumin incorporated poly(butylene adipate-co-terephthalate) film with improved water vapor barrier and antioxidant properties. *Materials*.

[6] Roy S., Rhim J. W. (2021). Antioxidant and antimicrobial poly(vinyl alcohol)-based films incorporated with grapefruit seed extract and curcumin. *Journal of Environment Chemical Engineering*.

[7] Lailatussifa R., Husni A., Isnansetyo A. (2017). Antioxidant activity and proximate analysis of dry powder from brown seaweed *Sargassum hystrix*. *Jurnal Perikanan Universitas Gadjah Mada*.

[8] Tuo K., Beourou S., Toure A. O. (2015). Antioxidant activities and estimation of the phenols and flavonoids content in the extracts of medicinal plants used to treat malaria in Ivory Coast. *International Journal of Current Microbiology and Applied Sciences*.

[9] Huang C. Y., Wu S. J., Yang W. N., Kuan A. W., Chen C. Y. (2016). Antioxidant activities of crude extracts of fucoidan extracted from *Sargassum glaucescens* by a compressional-puffing-hydrothermal extraction process. *Food Chemistry*.

[10] Alboofetileh M., Rezaej M., Tabarsa M. (2019). Effect of different non-conventional extraction methods on the antibacterial and antiviral activity of fucoidans extracted from *Nizamuddinia zanardinii*. *Journal of Biological Macromolecules*.

[11] Li B., Lu F., Wei X., Zhao R. (2008). Fucoidan: structure and bioactivity. *Molecules*.

[12] Senthilkumar K., Ramajayam G., Venkatesan J., Kim S., Ahn B. (2017). Biomedical applications of fucoidan, seaweed polysaccharides. *Seaweed Polysaccharides*.

[13] Sanjeewa K. K. A., Jeon Y. (2021). Fucoidans as scientifically and commercially important algal polysaccharides. *Marine Drugs*.

[14] Kadam S. U., Tiwari B. K., Smyth T. J., Odonell C. P. (2015). Optimization of ultrasound assisted extraction of bioactive components from brown seaweed *Ascophyllum nodosum* using response surface methodology. *Ultrasonics Sonochemistry*.

[15] Sinurat E., Maulida N. N. (2018). Pengaruh hidrolisis fukoidan terhadap aktivitasnya sebagai antioksidan. *Jurnal Pascapanen dan Bioteknologi Kelautan dan Perikanan*.

[16] Wang C. Y., Wu T. C., Hsieh S. L., Tsai Y. H., Yeh C. W., Huang C. Y. (2015). Antioxidant activity and growth inhibition of human colon cancer cells by crude and purified fucoidan preparations extracted from *Sargassum cristaefolium*. *Journal of Food and Drug Analysis*.

[17] Suhaila K., Husni A., Sinurat E. (2019). Characteristic and antioxidant activity of fucoidan from the brown seaweed *Sargassum hystrix*. *AACL Bioflux*.

[18] Kurnialahi I. D., Husni A., Sinurat E., Isnansetyo A. (2020). Antioxidant activity of tropical seaweed *Sargassum muticum* fucoidan. *AACL Bioflux*.

[19] Fernandez N. F., Garcia M. L., Munoz M. J. G., Vilarino J. M. L., Bominguez H. (2017). Ultrasound-assisted extraction of fucoidan from *Sargassum muticum*. *Journal of Applied Phycology*.

[20] Sinurat E., Kusumawati R. (2017). Optimization of crude fucoidan extraction methods from brown seaweed *Sargassum binderi* Sonder. *Jurnal Pascapanen dan Bioteknologi Kelautan dan Perikanan*.

[21] Kordjazi M., Etemadian Y., Shabanpour B., Pourashouri P. (2019). Chemical composition antioxidant and antimicrobial activities of fucoidan extracted from two species of brown seaweeds (*Sargassum ilicifolium* and *S. angustifolium*) around Qeshm Island. *Iranian Journal of Fisheries Sciences*.

[22] Purbomartono C., Isnansetyo A., Murwantoko T. (2019). Dietary fucoidan from *Padina boergesenii* to enhance non-specific immune of catfish (Clarias sp.). *Jurnal Biology Science*.

[23] Palanisamy S., Vinosha M., Marudhupandi T., Rajasekar P., Prabhu N. M. (2017). Isolation of fucoidan from *Sargassum polycystum* brown algae: structural characterization, *in vitro* antioxidant and anticancer activity. *International Journal of Biological Macromolecules*.

[24] Zhao D., Xu J., Xu X. (2018). Bioactivity of fucoidan extracted from *Laminaria japonica* using a novel procedure with high yield. *Food Chemistry*.

[25] Dubois M., Gilles K. A., Hamilton J. K., Rebers P. A., Smith F. (1956). Colorimetric method for determination of sugars and related substances. *Analytical Chemistry*.

[26] Dodgson K. S., Price R. G. (1962). A note on the determination of the ester sulphate content of sulphated polysaccharides. *Biochemical Journal*.

[27] Vagdevi H. M., Lokesh M. R., Gowdarshivannanavar B. C. (2012). Synthesis and antioxidant activity of 3-substituted Schiff bases of quinazoline 2.4-diones. *International Journal of ChemTech Research*.

[28] Clarke G., Ting K. N., Wiart C., Fry J. (2013). High correlation of 2,2-diphenyl-1-picrylhydrazyl (DPPH) radical scavenging, ferric reducing activity potential and total phenolics content indicates redundancy in use of all three assays to screen for antioxidant activity of extracts of plants from the Malaysian rainforest. *Antioxidants*.

[29] Salma H., Sedjati S., Ridlo A. (2019). Aktivitas antioksidan fraksi etil asetat dari ekstrak metanol *Sargassum sp*. *Journal of Marine Research*.

[30] Ale M. T., Mikkelsen J. D., Meyer A. S. (2011). Important determinants for fucoidan bioactivity: a critical review of structure-function relations and extraction methods for fucose-containing sulfated polysaccharides from brown seaweeds. *Marine Drugs*.

[31] Puspantari W., Kusnandar F., Lioe H. N., Laily N. (2020). Penghambatan fraksi fukoidan rumput laut cokelat (*Sargassum polycystum* dan *Turbinaria conoides*) terhadap *α*-amilase dan *α*-glukosidase. *Jurnal Pengolahan Hasil Perikanan Indonesia*.

[32] Lim S. J., Mustapha W., Yusof M., Mamod S. (2014). Isolation and antioxidant capacity of fucoidan from selected Malaysian seaweeds. *Jurnal Food Hydrocolloids*.

[33] Wang S., Huang C., Chen C. (2020). Structure and biological activity analysis of fucoidan isolated from *Sargassum siliquosum*. *American Chemical Society Omega*.

[34] Zuraida S., Sajuthi D., Suparto I. H. (2017). Fenol, flavonoid, dan aktivitas antioksidan pada ekstrak kulit batang pulai (*Alstonia scholaris* R.Br). *Jurnal Penelitian Hasil Hutan*.

[35] Panjinugroho F. D. (2016). *Pengaruh Temperatur dan Zat Aditif Asam Sitrat 20 ppm pada Pembentukan Kristal CaSO4 (Kalsium Sulfat)*.

[36] Sankari G., Kriahnamoorhty E., Jayakumaran S. (2010). Analysis of serum immunoglobulins using Fourier transform infrared spectral measurements. *Biology and Medicine*.

[37] Sinurat E., Rosmawaty P., Saepudin E. (2011). Extraction and activity test of fucoidan from brown seaweed (*Sargassum crassifolium*) as an anticoagulant. *Jurnal Pascapanen dan Bioteknologi Kelautan dan Perikanan*.

[38] Koh H. S. A., Lu J., Zhou W. (2019). Structure characterization and antioxidant activity of fucoidan isolated from *Undaria pinnatifida* grown in New Zealand. *Carbohydrates Polymers*.

[39] Kereh V. G., Kusnandar F. (2018). Karakteristik kimia ekstrak rumput laut serta kemampuannya menghambat bakteri *Salmonella sp*. *Jurnal Veteriner Desember*.

[40] Wang C., Liu X., Guo L. (2010). Two new natural ketoacid derivatives from *Sargassum pallidum*. *Chemistry of Natural Compounds*.

[41] Nurhidayati L., Fitriani Y., Abdillah S., Mumpuni E., Rafi M. (2020). Physicochemical properties and antioxidant activities of crude fucoidan extracted from *Sargassum cinereum*. *Jurnal Ilmu Kefarmasian Indonesia*.

